# COVID-19 as an effect modifier of the relationship between age and in-hospital survival in older patients admitted to an Italian Emergency Department

**DOI:** 10.1007/s40520-022-02115-x

**Published:** 2022-03-30

**Authors:** Alberto Zucchelli, Catello Sepe, Luca Tarozzi, Anna Garelli, Francesca Benedusi, Samuele Pignataro, Ciro Paolillo, Alessandra Marengoni

**Affiliations:** 1 Department of Clinical and Experimental Sciences, Università degli Studi di Brescia, viale Europa 11, 25123 Brescia, Italy; 2 Emergency Department Unit, ASST Spedali Civili, Brescia, Italy

**Keywords:** COVID-19 survival, Emergency Department, Older age

## Abstract

**Background:**

The SARS-CoV-2 pandemic modified how persons got into contact with emergency services, particularly during the first wave.

**Aim:**

The aim is to describe the characteristics of older persons with and without COVID-19 visiting the Emergency Department of a tertiary hospital and to investigate the impact of age on in-hospital survival in the two groups.

**Methods:**

Patients older than 70 years were followed-up till discharge or in-hospital death. Cox regression models stratified by COVID-19 diagnosis were used to investigate survival.

**Results:**

Out of 896 patients, 36.7% had COVID-19. Those without COVID-19 were older and affected by a higher number of chronic conditions but exhibited lower mortality (10.5 vs 48.1%). After the adjustment, age was associated with mortality only among those with COVID-19.

**Discussion:**

COVID-19 modified the relationship between older age and in-hospital survival: whether this finding is explained by other biological vulnerabilities or by a selection of treatments based on age should be further investigated.

**Supplementary Information:**

The online version contains supplementary material available at 10.1007/s40520-022-02115-x.

## Introduction:

SARS-CoV-2 pandemic had a huge impact on health systems worldwide and older persons paid the highest death toll [1], whether affected by COVID-19 or not [2]. Indeed, older age was identified, since the first reports about SARS-CoV-2 infection, as one of the most important risk factors for mortality in COVID-19. Excess mortality during the pandemic was shown to be increased among older persons, irrespectively of SARS-CoV-2 infection diagnosis. In addition, the significant work burden posed on hospital and emergency services by the pandemic, as well as local and global policies, modified the way persons got into contact with health systems [3–5]. This condition was further strengthened by forms of ageism that took place during the pandemic [6]. However, information about the characteristics of older persons admitted to Emergency Room (ER) during the first wave of the pandemic, irrespectively of COVID-19 diagnosis, are seldom reported. Furthermore, data about the impact of older age on survival in persons with and without COVID-19 admitted to hospitals during the worst days of the pandemic are still lacking.

To quantify the different weight that chronological age had on in-hospital mortality in persons with and without COVID-19 may help to better understand the challenges modern health systems had to face during to pandemic and to highlight possible pitfalls that should be addressed in the future.

Thus, we aimed (1) to describe and compare the characteristics of older persons with and without COVID-19 visiting the ER of one of the main hospitals in Northern Italy during the first wave of the pandemic and (2) to investigate the different impact of chronological age on in-hospital survival among older persons hospitalized with and without COVID-19.

## Methods

### Study population and data collection

All persons aged at least 70 years and admitted to the ER of Spedali Civili (Brescia), one of the main hospitals in Northern Italy, during the “first COVID-19 wave” (24th of February–11th of April 2020) were considered for inclusion in the present study. Patients who were transferred to other hospitals from the ER or after hospital admission were included in the descriptive analyses (aim 1) but were excluded from the survival analyses (aim 2), because we were not able to retrieve in-hospital outcomes. Demographics, medical history, chronic therapy information, signs, symptoms, blood, and imaging exams were retrieved from the informatic system in use in the ER. This information was entered by nurses and physicians according to clinical necessities at the time of ER visit. ER outcomes (e.g.: hospital admission, discharge, transferral to other hospitals, or death) were retrieved from the same informatic system. For patients admitted to our hospital, outcome (e.g.: discharge, transferral, or death) and length of stay was retrieved from the informatic letter of discharge. SARS-CoV-2 infection diagnosis was based on either (1) a positive polymerase chain reaction (PCR) test on any nasal and/or pharyngeal swab performed during the ER stay (81.9% of diagnosis) or (2) a clinical diagnosis, based on history, signs, symptoms, and radiological findings, posed by the attending physician. All study participants were categorized as either affected by COVID-19 or by other conditions using information retrieved from both ER data and discharge letters. Benzodiazepines, antidepressants, antipsychotics, antiepileptics, and anti-dementia drugs were grouped in “neuropsychiatric therapy”. This study was approved by the local ethical committee.

### Statistical analyses

Patients’ characteristics were described using median and interquartile range (IQR) or count and proportion, as appropriate. Univariate differences between patients affected by COVID-19 and the others were investigated using Mann–Whitney *U* test or chi-squared test, as appropriate. To investigate the possible role of COVID-19 as an effect modifier of the relationship between age and survival, we employed Cox regression models (both unadjusted and adjusted), stratified by COVID-19 diagnosis. Due to the presence of non-proportional hazards, the observation time was split in three intervals: the time cut-offs were chosen upon visual inspection of scaled Schoenfeld residual plots. An analysis evaluating the presence of a multiplicative interaction was conducted in all time intervals. The proportional hazard assumption was tested and satisfied for each model in each time interval. All analyses were conducted with R version 4.0.5 (R Foundation for Statistical Computing, Vienna—Austria) with an alpha-level of 0.05.

## Results

In total, 1141 patients were considered for inclusion in this study. Patients’ characteristics are described in Table 1. The median age was 80.8 years and 54.1% were males. In total, 475 patients were diagnosed with COVID-19, whereas 666 were considered free of SARS-CoV-2 infection. The latter group, compared with the former, exhibited a higher proportion of persons 85 + year-old (33.2% vs 22.1%), an increased median number of chronic conditions (4.0 vs 3.0), a higher prevalence of atrial fibrillation (20.1% vs 14.5%), cognitive impairment (14.0% vs 9.1%), falls (19.8% vs 9.9%), and of chronic prescription of neuropsychiatric drugs (18.6% vs 12.6%). Conversely, patients with a COVID-19 diagnosis exhibited higher median C-reactive protein concentration (CRP—56.6 vs 6.0 mg/dL) and lower median peripheral blood oxygen saturation (SpO2—94.0 vs 97.0%), as well as a higher proportion of fever (70.5% vs 9.6%) and dyspnoea (44.8% vs 10.7%) as presentation signs and symptoms, when compared with those without COVID-19.

**Table 1:** Characteristics of the study participants, stratified by COVID-19 diagnosis

	Whole population *N* = 1141	COVID-19 = no *N* = 666	COVID-19 = yes *N* = 475	*P*
Age, years (median (IQR))	80.8 (74.5, 85.7)	81.5 (75.3, 86.8)	79.9 (73.8, 84.6)	0.001
Age 85 + (%)	326 (28.6)	221 (33.2)	105 (22.1)	< 0.001
Male sex (%)	617 (54.1)	335 (50.3)	282 (59.4)	0.003
Heart failure (%)	119 (10.4)	72 (10.8)	47 (9.9)	0.689
Atrial fibrillation (%)	203 (17.8)	134 (20.1)	69 (14.5)	0.018
COPD (%)	107 (9.4)	65 (9.8)	42 (8.8)	0.674
Cognitive impairment (%)	136 (11.9)	93 (14.0)	43 (9.1)	0.015
Chronic kidney disease (%)	185 (16.2)	118 (17.7)	67 (14.1)	0.121
Number chronic conditions (%)	4.0 (2.0, 6.0)	4.0 (2.0, 6.0)	3.0 (2.0, 5.0)	< 0.001
Chronic neuropsychiatric therapy (%)	184 (16.1)	124 (18.6)	60 (12.6)	0.009
Chronic steroid therapy (%)	62 (5.4)	39 (5.9)	23 (4.8)	0.540
Fever (%)	399 (35.0)	64 (9.6)	335 (70.5)	< 0.001
Dyspnoea (%)	284 (24.9)	71 (10.7)	213 (44.8)	< 0.001
Smell and taste alterations (%)	16 (1.4)	1 (0.2)	15 (3.2)	< 0.001
Hyperactive delirium (%)	35 (3.1)	11 (1.7)	24 (5.1)	0.002
Falls (%)	179 (15.7)	132 (19.8)	47 (9.9)	< 0.001
SPO_2_, % (median (IQR))	96.0 (93.0, 98.0)	97.0 (95.0, 98.0)	94.0 (90.0, 96.0)	< 0.001
C-reactive protein, MG/DL (median (IQR))	25.9 (4.2, 83.2)	6.0 (1.7, 27.4)	56.6 (25.0, 117.8)	< 0.001
Total length of stay (ER + ward admission), days (median (IQR))	3.9 (0.4, 9.6)	1.9 (0.2, 6.4)	7.0 (2.1, 13.7)	< 0.001
Total in-hospital mortality (ER + ward), (%)	191 (16.8)	43 (6.5)	148 (31.1)	< 0.001
ER length of stay, hours (median (IQR))	5.4 (2.3, 12.8)	3.3 (1.6, 6.4)	12.1 (5.6, 22.9)	< 0.001
ER outcome (%)				< 0.001
Death	32 (2.8)	12 (1.8)	20 (4.2)	
Discharge	303 (26.6)	260 (39.2)	43 (9.1)	
Surgical ward admission	210 (18.5)	174 (26.2)	36 (7.6)	
Medical ward admission	513 (45.1)	192 (29.0)	321 (67.6)	
ICU admission	18 (1.6)	10 (1.5)	8 (1.7)	
Non-specified	10 (0.9)	5 (0.8)	5 (1.1)	
Transferred to other hospitals	52 (4.6)	10 (1.5)	42 (8.8)	
Length of stay since ward admission, days (median (IQR))	3.4 (0.0, 9.2)	1.4 (0.0, 6.1)	6.4 (0.7, 13.0)	< 0.001
Hospitalization outcome (%)				< 0.001
Death	159 (21.1)	31 (8.0)	128 (34.6)	
Discharge	405 (53.7)	266 (69.3)	139 (37.6)	
Not available	40 (5.3)	18 (4.7)	22 (5.9)	
transferred	150 (19.9)	69 (18.0)	81 (21.9)	

*IQR* interquartile range, *COPD* chronic obstructive pulmonary disease, *SpO*_*2*_ peripheral haemoglobin oxygen saturation, *ICU* intensive care unit. Missing: 283 for C-reactive protein, 127 for SpO_2_

The median ER length of stay was more than 12 h for those with COVID-19 whereas, for the other group, it was around 3 h. The discharge rates from the ER were 9.1% and 39.2% for the patients with and without COVID-19, respectively. The share of patients admitted to surgical and medical wards was similar among patients without COVID-19, whereas the majority of those diagnosed with a SARS-CoV-2 infection was admitted to medical wards. The proportion of patients admitted to the Intensive Care Unit was similar between the two groups. The 8.8% of patients with SARS-CoV-2 were transferred from the ER to other hospitals, whereas only the 1.5% of those without COVID-19 was transferred. ER mortality was more than two times higher among patients with COVID-19 (4.2%) than in the group of those without such diagnosis (1.8%).

For the survival analyses, we excluded those patients who were transferred to other hospitals, whose vital status after the hospitalization was not available or with missing information about hospital length (*N* = 245). The characteristics of this subsample were similar to the ones of the whole study population, as shown in Table S1.

Overall, in-hospital mortality was 16.8% (*N* = 191): 32 persons died in the ER, whereas the remaining died after being admitted to a hospital ward. Considering only the patients admitted to a hospital ward (*N* = 561), in-hospital mortality was 48.1% (128 out of 266 admitted) and 10.5% (31 out of 295 admitted) for patients with and without COVID-19, respectively. For the survival analyses, we considered all available follow-up time (since ER admission to 74.4 days, the maximum follow-up time): this period was split in three intervals, using day 1 and day 7 as cut-offs (i.e.: ER admission to day 1, day 1 to day 7, and day 7 to end of follow-up). As shown in Table 2, among those without COVID-19, age was associated with an increased risk of mortality only in the third time interval (HR = 1.12, 95%CI = 1.00–1.26): however, after adjusting the model for chronic conditions (model 1), the association disappeared. Such results were confirmed after the further inclusion in the model of CRP and SpO2 at admission (model 2). Conversely, among those with a COVID-19 diagnosis, age was associated with an increased risk of death in the first two time intervals, even after complete adjustment (model 2 HR—95%CI = 1.15—1.05, 1.26 for the first interval and 1.09—1.04–1.13 for the second interval). A trend for interaction was detected for the first and second time intervals (p for day 1 = 0.057, p for day 1–day 7 = 0.052, p for day 7–end of follow-up = 0.858).

**Table 2:** Hazard ratios (HR) and 95% confidence intervals (95% CI) for age (1-year increase) for in-hospital mortality

	Without COVID-19				With COVID-19			
	N events / at risk	Model 0	Model 1	Model 2	N events / at risk	Model 0	Model 1	Model 2
		Age HR (95% CI)				Age HR (95% CI)		
Admission–day 1	19/567	1.00 (0.94–1.07)	0.99 (0.92–1.06)	1.08 (0.98–1.19)	18/329	1.12 (1.05–1.20)*	1.12 (1.04–1.20)*	1.15 (1.05–1.26)*
Day 1–day 7	15/287	1.04 (0.97–1.12)	1.02 (0.95–1.10)	1.08 (0.98–1.18)	68/276	1.09 (1.06–1.13)*	1.09 (1.05–1.13)*	1.09 (1.04–1.13)*
Day 7–day 90	9/108	1.12 (1.00–1.26)*	1.10 (0.98–1.23)	1.10 (0.99–1.22)	62/167	1.03 (0.99–1.07)	1.02 (0.98–1.07)	1.02 (0.98–1.06)

Analyses are stratified for COVID-19 diagnosis

Model 0: unadjusted; Model 1: adjusted for heart failure, atrial fibrillation, cognitive decline, chronic kidney disease, diabetes mellitus; Model 2: model 1 + sex + peripheral blood oxygen saturation and C-reactive protein concentration at ER admission

*p < 0.05

## Discussion

In this study, we showed that older persons not infected with SARS-CoV-2 who visited the ER of one of the main hospitals in Northern Italy during the first wave of the pandemic were generally slightly older, more likely to be affect by chronic conditions and cognitive impairment in comparison with those admitted to the ER in the same period because of COVID-19. We have also shown that the relationship between chronological age and in-hospital mortality was modified by COVID-19.

Some reasons may, at least partially, explain the differences in clinical and demographic characteristics exhibited by patients with and without COVID-19 in our study. First, during the worst days of the pandemic, persons living in nursing homes in Italy had a lower probability to receive a SARS-CoV-2 diagnosis and to be hospitalized if affected by COVID-19 [7, 8], in comparison with those living in the community. For example, in a survey issued by the Italian Ministry of Health, out of 667 deaths among nursing home residents in Brescia between February and March 2020, only 33 (5%) had a positive SARS-CoV-2 test, but respiratory symptoms were reported in 418 (63%) of them [8]. Furthermore, during the first wave, in North-eastern Italy, nursing home residents with poorer health (measured using the Multiprognostic Index) exhibited a significantly higher mortality risk in comparison with those characterized by better health. However, the hospitalization rate was similar between the two groups [9]. These conditions may have contributed to select a “younger” and “healthier” sample of persons affected by SARS-CoV-2 visiting the ER, composed by patients who were less likely to be affected by cognitive impairment and severe chronic conditions. In second place, a relative increase in the severity of ER diagnoses (other than COVID-19) was reported during the first wave of the pandemic in Italy [10]. It is plausible that persons who sought emergency care in that period for reasons other than COVID-19 were more likely to be affected by conditions that could not be treated at home, such as acute diseases needing urgent surgical (including orthopaedic) or medical treatment. It is possible that this situation led to the selection of a sample of patients without COVID-19 who more likely to be at higher risk for such conditions (i.e.: older and with a higher burden of chronic diseases).

Despite patients without COVID-19 showed a worse pre-morbid health status, they exhibited significantly lower in-hospital mortality than persons with COVID-19. As expected and largely reported by the literature [11], the inclusion of chronic conditions in the model minimized the impact of older age on survival: this finding confirms that biological age, rather than chronological one, is the main drive of in-hospital mortality. However, such paradigm held only in the subsample of persons admitted to hospital for reasons other than COVID-19, in our study. Among patients infected by SARS-CoV-2, we found that older age was associated with mortality even after adjusting for chronic conditions and (possibly over-adjusting) for disease severity at ER presentation. The impact of older age on in-hospital mortality in COVID-19 was reported since the first studies about the SARS-CoV-2 pandemic. However, age seemed to be associated with in-hospital mortality beyond proxies of biological age: in a previous work, we found that, even after taking into consideration multimorbidity and frailty (measured by the Clinical Frailty Scale), age was still associated with in-hospital mortality among patients admitted to a geriatric ward during the first wave of the pandemic [12]. This issue was confirmed in studies using other measures of frailty (i.e.: frailty index) [13]. It is possible that some biological vulnerabilities, not captured by current frailty and multimorbidity constructs but associated with older age, explain the association between chronological age and COVID-19 in-hospital mortality. However, it is also possible that the profound impact that the pandemic had on health systems and management (e.g.: saturation of intensive care beds) led to a selection of treatments based on chronological age, configuring a condition of ageism [14]. Furthermore, we found that ICU admissions were low (lower than 2%) for both patients with and without COVID-19: future studies should investigate whether younger age played a possible role as admission criterion for intensive care, irrespective of COVID-19 diagnosis, during the first wave of the pandemic.

Our study should be read in light of some limitations. In first place, we have only analysed data about in-hospital mortality and we were not able to retrieve medium- or long-term mortality information. In second place, due to the high work burden that ER physician experienced, data about pre-morbid functional status and frailty were not collected and we were not able to take into consideration this information in our analyses.

## Conclusion

COVID-19 modified the relationship between older age and in-hospital mortality. Future studies should highlight whether this finding is related to the biological vulnerabilities of patients or to the impact of the pandemic on health systems.

## Supplementary Information

Below is the link to the electronic supplementary material.

Supplementary file1 (DOCX 18 KB)
