# Supplementary material for: COVID-19 as an effect modifier of the relationship between age and in-hospital survival in older patients admitted to an Italian Emergency Department
Source: Aging Clin Exp Res. 2022 Mar 30;34(5):1195–200. doi: 10.1007/s40520-022-02115-x (PMC8966861; doi:10.1007/s40520-022-02115-x)
Supplement: Supplementary file 1 — Supplementary file1 (DOCX 18 KB) [file 40520_2022_2115_MOESM1_ESM.docx]

Table 1: characteristics of the study population

|  | Whole population  N = 896 | COVID-19 = No N = 567 | COVID-19 = Yes N = 329 | p |
| --- | --- | --- | --- | --- |
| Age, years (median (IQR)) | 80.7 (74.4, 85.7) | 81.2 (75.0, 86.4) | 80.0 (73.7, 84.8) | 0.051 |
| Age 85+ (%) | 255 (28.5) | 176 (31.0) | 79 (24.0) | 0.030 |
| Male sex (%) | 490 (54.7) | 286 (50.4) | 204 (62.0) | 0.001 |
| Heart failure (%) | 99 (11.0) | 61 (10.8) | 38 (11.6) | 0.800 |
| Atrial fibrillation (%) | 155 (17.3) | 111 (19.6) | 44 (13.4) | 0.023 |
| COPD (%) | 79 (8.8) | 50 (8.8) | 29 (8.8) | 1.000 |
| Cognitive impairment (%) | 100 (11.2) | 73 (12.9) | 27 (8.2) | 0.042 |
| Chronic kidney disease (%) | 147 (16.4) | 97 (17.1) | 50 (15.2) | 0.515 |
| Number chronic conditions (%) | 4.0 (2.0, 6.0) | 4.0 (2.0, 6.0) | 3.0 (2.0, 5.0) | <0.001 |
| Chronic neuropsychiatric therapy (%) | 131 (14.6) | 100 (17.6) | 31 (9.4) | 0.001 |
| Chronic steroid therapy (%) | 50 (5.6) | 34 (6.0) | 16 (4.9) | 0.575 |
| Fever (%) | 279 (31.1) | 55 (9.7) | 224 (68.1) | <0.001 |
| Dyspnoea (%) | 216 (24.1) | 56 (9.9) | 160 (48.6) | <0.001 |
| Smell and taste alterations (%) | 14 (1.6) | 1 (0.2) | 13 (4.0) | <0.001 |
| Hyperactive delirium (%) | 26 (2.9) | 7 (1.2) | 19 (5.8) | <0.001 |
| Falls (%) | 151 (16.9) | 116 (20.5) | 35 (10.6) | <0.001 |
| SpO_2_, % (median (IQR)) | 96.0 (94.0, 98.0) | 97.0 (95.0, 98.0) | 94.0 (90.0, 96.0) | <0.001 |
| C-reactive protein, mg/dL (median (IQR)) | 22.9 (3.5, 83.2) | 5.6 (1.7, 26.3) | 58.5 (24.7, 125.0) | <0.001 |
| Total length of stay (ER + ward admission), days (median (IQR)) | 3.4 (0.3-8.6) | 1.1 (0.2-5.5) | 7.2 (2.7-13.5) | <0.001 |
| Total in-hospital mortality (ER + ward), (%) | 191 (21.3) | 43 (7.6) | 148 (45.0) | <0.001 |
| ER length of stay, hours (median (IQR)) | 5.0 (2.2, 11.5) | 3.3 (1.7, 6.4) | 11.7 (5.1, 22.3) | <0.001 |
| ER outcome (%) |  |  |  | <0.001 |
| Death | 32 (3.6) | 12 (2.1) | 20 (6.1) |  |
| Discharge | 303 (33.8) | 260 (45.9) | 43 (13.1) |  |
| Surgical ward admission | 169 (18.9) | 145 (25.6) | 24 (7.3) |  |
| Medical ward admission | 376 (42.0) | 140 (24.7) | 236 (71.7) |  |
| ICU admission | 11 (1.2) | 7 (1.2) | 4 (1.2) |  |
| Non-specified admission | 5 (0.6) | 3 (0.5) | 2 (0.6) |  |
| Length of stay since ward admission, days (median (IQR)) | 6.4 (3.4, 12.3) | 5.2 (2.9, 9.0) | 8.3 (4.5, 15.0) | <0.001 |
| In-hospital mortality since ward admission (%) | 159 (28.3) | 31 (10.5) | 128 (48.1) | <0.001 |

Abbreviations: IQR = interquartile range; COPD = chronic obstructive pulmonary disease; SpO_2_ = peripheral haemoglobin oxygen saturation; ICU = intensive care unit. *Multimorbidity* is defined as being affected of at least two chronic conditions.
Missing: 153 for N. of chronic conditions (50 and 103 for patients with and without COVID-19), 251 for C-reactive protein (36 and 215 for patients with and without COVID-19), 105 for SpO_2_ (26 and 79 for patient with and without COVID-19)
